# Pancreatic Cancer: A Review of Risk Factors

**DOI:** 10.3390/life14080980

**Published:** 2024-08-05

**Authors:** Raluca Roxana Grigorescu, Ioana Alexandra Husar-Sburlan, Cristian Gheorghe

**Affiliations:** 1 Gastroenterology Department, “Sfanta Maria” Hospital, “Carol Davila” University of Medicine and Pharmacy, 020021 Bucharest, Romania; 2 Gastroenterology Department, “Sfanta Maria” Hospital, 011172 Bucharest, Romania; 3 Center for Digestive Disease and Liver Transplantation, Fundeni Clinical Institute, “Carol Davila” University of Medicine and Pharmacy, 020021 Bucharest, Romania

**Keywords:** pancreatic cancer, risk factors, obesity, microbiota, diabetes

## Abstract

Pancreatic adenocarcinoma is one of the most lethal types of gastrointestinal cancer despite the latest medical advances. Its incidence has continuously increased in recent years in developed countries. The location of the pancreas can result in the initial symptoms of neoplasia being overlooked, which can lead to a delayed diagnosis and a subsequent reduction in the spectrum of available therapeutic options. The role of modifiable risk factors in pancreatic cancer has been extensively studied in recent years, with smoking and alcohol consumption identified as key contributors. However, the few screening programs that have been developed focus exclusively on genetic factors, without considering the potential impact of modifiable factors on disease occurrence. Thus, fully understanding and detecting the risk factors for pancreatic cancer represents an important step in the prevention and early diagnosis of this type of neoplasia. This review reports the available evidence on different risk factors and identifies the areas that could benefit the most from additional studies.

## 1. Introduction

The vast majority of pancreatic cancers arise from the exocrine component and are classified as pancreatic adenocarcinoma, referred to in the text as pancreatic cancer (PC). It should be noted that there are other, less common histological subtypes, including invasive intraductal papillary mucinous neoplasm (IPMN), adenosquamous carcinoma, invasive mucinous cystic neoplasm (MCN), acinar cell and squamous cell carcinoma, and invasive solid pseudopapillary tumor. In less than 10% of cases, the condition can develop from endocrine cells and form neuroendocrine tumors, which have a more favorable prognosis [1]. PC has the worst prognosis and a very high mortality [2], with almost as many deaths as new cases per year. The delay in diagnosis is often due to its non-specific and insidious symptoms, such as middle epigastric pain that sometimes radiates to the back, weight loss, nausea, fatigue, and, if the location of the tumor is in the pancreatic head, jaundice. The lifetime risk of developing this neoplasia is 0.91–1.6% [2,3,4]. The incidence has doubled in the last two decades and is up to four times higher in countries with a higher Human Development Index [5,6]. Less than 20% of patients have a surgically resectable tumor at diagnosis, with a median survival of 12.6 months. In contrast, for those for whom surgery is no longer an option, the median survival rate is 3.5 months [7]. Incidentally discovered PC is associated with longer survival than the one diagnosed due to signs and symptoms [8].

In the last years, according to data indicated by the Surveillance, Epidemiology, and End Results (SEER) program, the incidence of pancreatic cancer represented 3.2% of all cancer cases, with an estimated mortality of 8.2% of all deaths determined by any cancer [3]. It was estimated that by the year 2030, PC incidence will increase to 15.1 per 100,000 [9] and become the second leading cause of cancer-related death [10], and the incidence will further increase by an average of 1.1% per year, up to 18.6% in 2050 [9].

It has been suggested that the sequence in sporadic pancreatic carcinoma is characterized by the change in the normal duct epithelium to ductal hyperplasia and invasive ductal adenocarcinoma. During this period, a sequence of genetic alterations may occur: first in KRAS (Kirsten-rat sarcoma) and Her-2, followed by p-16 alteration, and finally, in p53, DPC4 and BRCA2, and other tumor suppressor genes [11,12]. The presence of KRAS mutation in pancreatic juice has been frequently observed in patients with pancreatic cancer (80%) and also in patients with chronic pancreatitis (CP) before the development of PC [13,14,15,16], suggesting that it may be useful in identifying CP patients who are at a higher risk of developing cancer. On the contrary, studies on the presence of KRAS mutation performed on stools and serum samples found that it can also be present in cases of benign pancreatic diseases, thus compromising its utility as a screening tool [15,17].

The aim of this study is to review the current literature on the role of several modifiable and non-modifiable risk factors in the pathogenesis and progression of prostate cancer (PC) and to identify potential strategies for preventing the onset of this disease. Exposure to modifiable and non-modifiable risk factors may independently or jointly contribute to the occurrence of pancreatic cancer. The detection of risk factors and precursor lesions will help identify the individuals at a higher risk for this neoplasia, improve prevention efforts to reduce exposure to certain factors, reduce the increasing incidence, and help in the early detection of this neoplasia.

## 2. Methods

The study included original research papers encompassing a range of methodologies, including retrospective and prospective cohort studies, clinical case-control studies, and systematic reviews. Publications such as technical reports, editor responses, narrative reviews, in silico studies, applied scientific posters, research proposals, and conference abstracts were excluded. Additionally, articles not in English and those unrelated to adenocarcinoma pancreatic risk factors were excluded.

The research was gathered through PubMed and the Google Academic Database. The searches covered a period of 10 years prior to September 2023. Articles were identified through the use of keywords such as “risk factors”, “pancreatic cancer”, and “pancreatic adenocarcinoma”. Subsequently, each risk factor, both modifiable and non-modifiable, was searched individually.

Two independent reviewers (RRG, IHD) conducted a comprehensive text and abstract screening and selected the relevant studies.

## 3. Risk Factors

**Figure 1:**
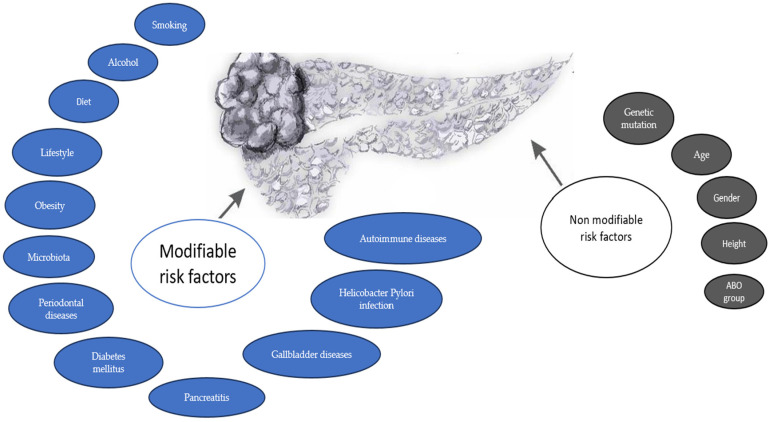
Modifiable and non-modifiable risk factors in pancreatic cancer.

Risk factors may be divided into non-modifiable risk factors, like age, sex, blood type, and genetic susceptivity, and modifiable risk factors, like diet, obesity, infections, and exposure to different chemicals and drugs (Figure 1).

### 3.1. Non-Modifiable Risk Factors

#### 3.1.1. Age

Pancreatic cancer can develop at any age but frequently occurs in patients aged between 60 and 80 years [10,18,19,20]. In the last decades, life expectancy has increased steadily at a rate of 2.5 years per decade, reaching over 84 years in Japan [21]. Globally, over 11% of the world’s population is over 60 years of age, and a doubling of this percentage is projected by 2050 [22]. Given the peak incidence of pancreatic cancer and the aging rate of the population, this will lead to an increase in the number of cases, thus burdening the health system.

The link between aging and an increased incidence of neoplasia may be explained by the gradual aging-associated dysfunction of the mitochondrial electron transport chain, resulting in the increased production of superoxides with an accumulation of oxygen radicals and increased oxidative stress over the years, leading to damage to multiple cellular elements and determining phenotype changes [22].

Early onset pancreatic cancer (EOPC) is a term used to define patients diagnosed with pancreatic tumors before the age of 50 [18,23]. The relative frequency of EOPC ranges between 5 and 18% in different studies [23,24,25]. The diagnosis is frequently made at more advanced stages than for late-onset pancreatic cancer (LOPC). However, despite the stage, there is a tendency to administer significantly more treatment or to perform more surgical interventions for those with EOPC. The prognosis remains reserved, especially in the case of operated patients. In the case of unoperated ones, although survival remained dismal, no significant difference was observed compared to that of patients with LOPC [18].

#### 3.1.2. Sex

The rates for PC, including EOPC, are consistently higher in men than in women [26,27]. The reason why there is a difference between the sexes is not entirely known, but it could be explained by a lower exposure of women to the modifiable risk factors.

#### 3.1.3. Height

Some studies stipulated that increased height may be associated with an increased risk of pancreatic cancer [28], with an RR of 1.81 (95% Cl, 1.31–2.52) [29], but others failed to find such an association [30,31]. The correlation between adult height and neoplasia, which was also observed in other cancers [32], could be explained by exposure to higher levels of growth factors during childhood and early adolescence.

#### 3.1.4. ABO Group

The ABO blood group antigens are encoded by one genetic locus on chromosome 9q34, which encodes three glycosyltransferases with different substrate specificities. Several studies have indicated that patients with blood type O appear to have a relatively lower risk of developing PC than other blood groups, particularly group A, which is significantly more common in affected patients [33,34,35]. It would seem that some glycosyltransferase enzymes may play a role in malignant cell immunosurveillance and intercellular adhesion during tumor genesis, but the mechanism for this association is not fully understood [36]. In the absence of a clear explanation, potentially attributable to augmented clearance and/or diminished secretion, it has been observed that antigen A is associated with diminished sICAM-1 concentrations [37]. This may lead to an increase in inflammation, which could further contribute to tumor growth and development. An association between PC and CagA-negative *Helicobacter pylori* (*H. pylori*) positivity has been demonstrated in one study among non-O blood type individuals but not for those with O blood type [38]. Conversely, a recent study confirmed an increased risk for those with non-0 blood type but failed to find a correlation with *H. pylori* infection status or CagA virulence status [39].

#### 3.1.5. Genetic Factors

**Figure 2:**
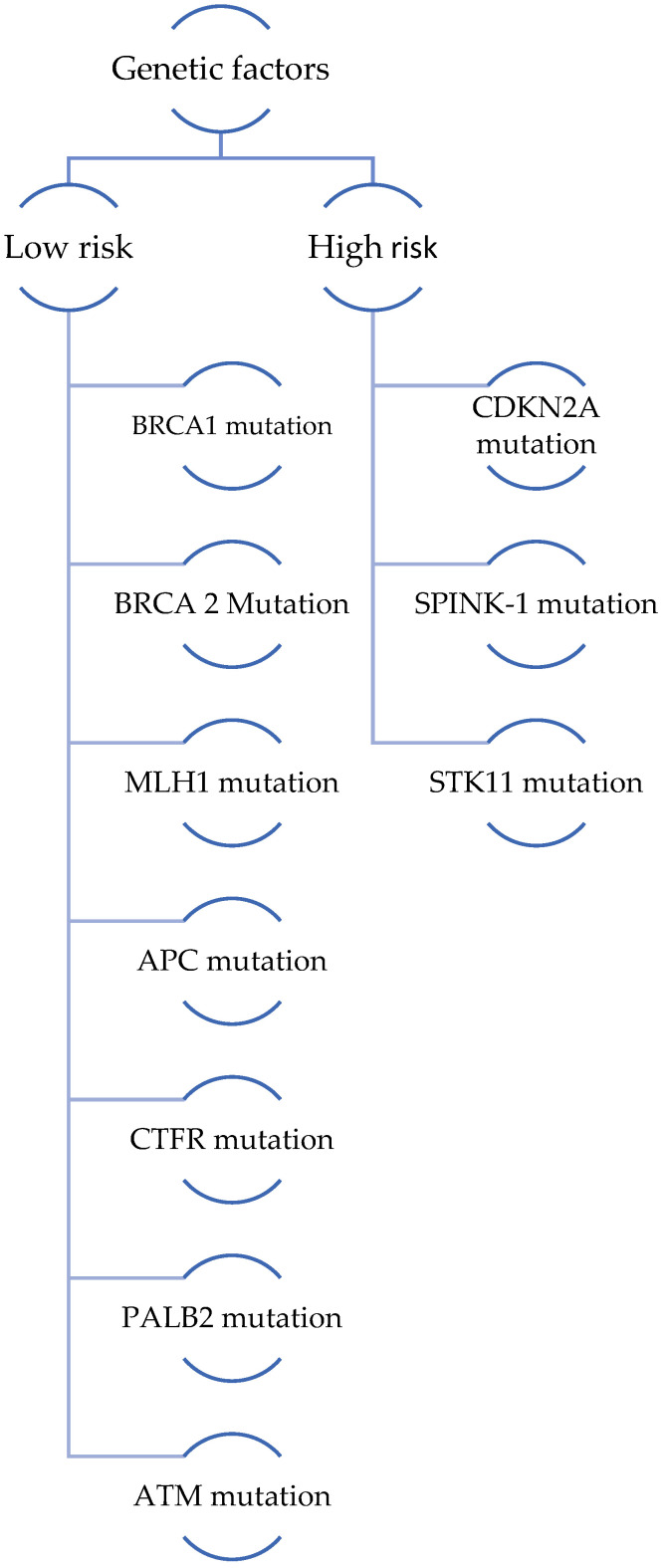
Genetic mutations grouped according to the risk of developing PC.

A hereditary component of PC has been observed in less than 10% of cases. The risk of developing pancreatic cancer varies depending on the specific genetic mutation present (Figure 2).

BRCA1 and BRCA2 are tumor suppressor genes that encode the proteins involved in repairing proteins that restore DNA double-stranded breaks via homologous recombination [40]. Mutations of these genes promote genomic instability and increase predisposition to malignant transformation and progression [41]. BRCA1 and 2 mutations have a general population occurrence rate between 1/300 and 1/800 [42], with a higher rate for the Ashkenazi Jewish ethnic group [43]. The penetrance is higher for female breast and ovarian cancer than for male breast cancer or PC [44]. Multiple studies have identified a risk up to threefold higher for BRCA1 mutation carriers in both sexes [43,45,46]. Mutations in BRCA2 have been found in up to 19% of familial PC and 7.3% of sporadic PC cases [47]. Identifying patients with BRCA mutation may represent an essential step in PC management, favoring early diagnosis and an increased surgical resection rate of PC. It was also found that these mutations could have implications for treatment options, leading to an increased response to platinum-based chemotherapy [48]. FANCC and FANCG mutations have been associated with EOPC [49,50].

Lynch syndrome (HNPCC) is the most common inherited colorectal cancer type. It is frequently associated with other types of cancer affecting various sites, such as the brain, pancreas, stomach, small intestine, urinary tract, endometrium, and ovary [51,52]. These patients have a germline mutation of genes encoding proteins involved in DNA mismatch repair: MSH2, MSH6, MLH1, PMS 1 and 2, and/or EPCAM [52], and they have an 8.6-fold increased risk of PC [53], with a lifetime risk of 1.3–4% [54]. The PC occurring in HNPCC patients frequently has a distinctive medullary appearance, which is otherwise a rare type of pancreatic adenocarcinoma and is associated with a better prognosis than conventional ductal adenocarcinoma [55,56,57]. Conversely, patients with the medullary PC phenotype are more likely to have a family history of cancer in first-degree relatives [56], suggesting a probable inherited susceptibility to HNPCC. It is essential to identify patients with PC and a pathogenic variant of the Lynch syndrome genes, as they may have tumors deficient in mismatched DNA repair and respond better to immunotherapy, as also suggested by Le et al. [58].

Familial adenomatous polyposis (FAP) is determined by mutations in the adenomatous polyposis coli (APC) tumor suppressor gene. It is characterized by the development of hundreds of colonic adenomatous polyps that progress to malignancy. It can also be associated with cancer in other sites such as the brain, thyroid, duodenum, pancreas, or hepatoblastoma [59]. Some studies stipulate that the risk of pancreatic cancer in this condition can be up to four times higher than in the general population (HR, 6.45; 95%CI, 2.02–20.64; *p* = 0.002) [60,61], thus raising awareness for updating active surveillance programs, even after prophylactic colectomy. However, these data may be misleading because some studies may have included patients with ampullary cancer due to misclassification, because the diagnosis of PC was not always histological, and a diagnosis based solely on imaging techniques may have confounded the two.

Peutz–Jeghers syndrome (PJS) is an autosomal dominant disease caused by mutations of the serine-threonine kinase 11 (STK11/LKB1) gene, a tumor suppressor gene that determines hamartomatous gastrointestinal polyposis and typical mucocutaneous pigmentation [62,63]. It is also associated with an increased risk of cancer of the esophagus, stomach, small intestine, colon, pancreas, pulmonary, breast, ovary, and uterus [64]. The relative risk for PC is increased to 76 (95%CI 36 to 160; *p* < 0.001) [65]. It is plausible that the risk of pancreatic cancer may be elevated in individuals with Peutz–Jeghers syndrome, akin to the observations made in the familial adenomatous polyposis population. This is potentially due to the difficulties in differentiating between pancreatic, distal bile duct, and ampullary cancers in some studies. Familial atypical mole melanoma pancreatic carcinoma syndrome (FAMMM-PC) is characterized by multiple malignant melanomas in first- and second-degree relatives and a 13 to 67-fold increased risk of pancreatic cancer, frequently with early onset (median age 55 years) [66,67,68,69]. It is caused by a germline mutation of p16INK4A (also known as CDKN2A or MTS1) [70,71]. CDKN2A is a gene implicated in cell proliferation. Parker et al. described that in cases of FAMMM, PC develops from normal duct epithelium to ductal hyperplasia to invasive ductal adenocarcinoma and suggested that patients with melanoma and CDKN2A mutation should be screened for pancreatic adenocarcinoma [72]. The identification of the CDKN2A mutation places patients at a high risk for pancreatic cancer, and their inclusion in screening programs improves the survival rate, as demonstrated by Vasen et al., who incorporated 19 patients with a CDKN2A mutation in such programs and found 13 patients with PC, 9 of them having a 5-year survival rate of 75% [73], higher than previously reported for patients with PC.

The SPINK1 gene encodes a serine protease inhibitor that inhibits active trypsin [74], and the PRSS1 gene encodes the prodigestive enzyme trypsinogen [75]. Mutation to PRSS1 can increase trypsin stability or trypsinogen autoactivation, favoring the development of chronic pancreatitis [76,77]. Mutation to SPINK1 and PRSS1 genes determine hereditary pancreatitis (HP) with a penetrance rate of 80% [78]. Recurrent episodes of pancreatitis determine chronic inflammation that decreases immunosurveillance and facilitates cell proliferation through Il-6 and 11, increasing the risk of PC [79]. Patients with hereditary pancreatitis have a 53-fold increase in the risk for PC (95%CI: 23–105) compared to the general population [80]. Smokers with hereditary pancreatitis develop the disease an average of 20 years before non-smokers [81].

Cystic fibrosis is determined by the mutation of the CFTR gene that regulates the flow of chloride ions across cell membranes. The mutation of this gene impairs ion transport and subsequently determines a chronic inflammation state in both respiratory and digestive systems [82]. Cystic fibrosis is associated with a 5.3-fold increased risk of PC, frequently with early onset [83]. This may be due to the increased viscosity of pancreatic juice, which may determine clogs, inflammation, and cell damage.

Ataxia-telangiectasia is caused by a homozygous mutation in the ATM gene that encodes a serine/threonine kinase involved in DNA repair [84]. It is characterized by progressive cerebellar ataxia, oculomotor apraxia, telangiectasias, increased sensitivity to ionizing radiation, and an increased risk of malignancies, such as breast and pancreatic neoplasia [84,85,86].

Based on the presence of a family history of PC and one of the above germline mutations, the International Cancer of the Pancreas Screening (CAPS) Consortium has developed a guideline to assist clinicians in the early diagnosis of this disease to improve survival. Screening should be made by MRI/MRCP or EUS, fasting serum glucose, and/or HbA1c at baseline and by adding CA 19–9 for a follow-up [87]. Considering that patients with at least one first-degree relative (FDR) and one second-degree relative (SDR) with PC are at an increased risk for this type of neoplasia, they should be included in screening programs at the age of 45–55 or at ten years younger than the youngest relative with PC [87]. The screening recommendations for those patients who are carriers of germline mutations are summarized in the table below (Table 1). The follow-up should be made yearly by MRI or EUS if no pancreatic abnormalities or low-risk features are detected. Although it was seen that smoking is a risk factor for progression, the interval is not shorter for this particular group. The role of surveillance of circulating tumor DNA in high-risk individuals still needs to be established.

#### 3.1.6. Preneoplasic Pancreatic Lesions

**Figure 3:**
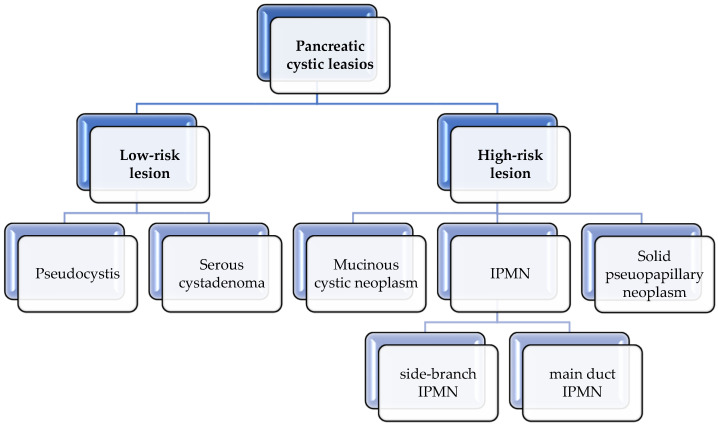
Pancreatic cystic lesions and the risk of PC.

Cystic lesions of the pancreas are tumor formations with varying degrees of malignant potential that can be classified as high risk or low risk (Figure 3). The risk of progression to pancreatic cancer (PC) varies depending on the relationship with the pancreatic duct. For instance, the risk of progression to PC in branch duct IPMN (BD-IPMN) is 19–30% [88], while in main duct IPMN (MD-IPMN), it is 40–60% [89].

There are clinical, radiological, and pathological features that may help in differentiating between various pancreatic lesions [90,91,92,93,94,95,96]. Although the MRI/MRCP is excellent for evaluating the location of pancreatic nodules, there is a high inter-observer variability regarding the risk stratification for IPMN [97]. Thus, because of the lack of reliable imaging and biomarkers that can help predict the development of some pancreatic cystic lesions, and also because there are even inconsistencies between various guidelines (e.g., Fukuoka criteria, AGA, ACR), the screening recommendations remain in the hands of the clinicians.

In recent years, novel techniques have been under study to help differentiate between pancreatic lesions, such as EUS-guided SF6 pancreatography (it can more accurately determine the communication between pancreatic cystic lesions and the pancreatic duct, with a sensitivity of 96.6% and specificity of 88.9% [98]) or PET using the ^68^Ga-labeled fibroblast activation protein inhibitor—^68^Ga-FAPI PET (it showed promising results in differentiating between high- and low-risk IPMN) [99]. In pancreatic cystic lesions, such as IMPN and MCN, the risk of PC development is higher for those with a male sex, increased age, smoking, obesity, and diabetes, including non-obese diabetes [100,101,102,103]. Regarding the link between IPMN and diabetes, it was found that patients with diabetes, especially those using insulin, have a higher incidence of BD-IPMN [100,104]. Surprisingly non-obese diabetes was not associated with an increased risk of IPMN, suggesting that these lesions do not secrete diabetogenic substances, like PC.

A nomogram taking into account the serum CEA and CA 19–9, the dimensions of the cyst, the presence of a thickened enhanced wall and an enhancing mural nodule, the main pancreatic duct (MPD) diameter, the presence of jaundice, and the prognostic nutrition index was found to have a high, over 80%, sensitivity and specificity for detecting high-risk IPMNs [105], but validation in a prospective study is needed. Another retrospective study also found that male sex, smoking, multifocality, diabetes, and recent weight loss were associated with developing worrisome features and high-grade dysplasia or cancer [106]. Worrisome features on imaging at presentation, the smoker status, and the presence of symptoms at diagnosis were associated with IPMN progression in a recent retrospective study [107]. Thus, patients diagnosed with pancreatic cysts have to be encouraged to cease smoking and maintain a normal BMI in order to reduce the risk of progression to neoplasia.

The value of CA 19–9 in screening programs is inconsistent, without a clear cut-off value. Although 37 kU/L increases the sensitivity, a higher value of 133 kU/L increases the specificity, avoiding the performance of multiple unnecessary screening tests or surgery [108]. Multiple studies found that patients with BD-IPMN develop worrisome futures or high-risk stigmata beyond the standard period for surveillance of 5 years and advise continuing surveillance after this period [109,110]. Studies on the psychological impact of PCN surveillance found higher anxiety levels and stress, with an overall reduced quality of life. All of the above should be considered when proposing a conservative approach that presumes regular clinic visits and individualized case-by-case visits [111].

### 3.2. Modifiable Risk Factors

#### 3.2.1. Smoking

Smoking can induce PC through the cumulative effect of tobacco-related carcinogens (heterocyclic amines and polycyclic aromatic hydrocarbons) that can produce mutation to both proto-oncogenes and tumor suppressor genes and through smoke-induced chronic inflammation [112]. Animal studies have suggested that nicotine itself also acts by inducing the secretion of interleukin 8 (IL-8) and the upregulation of the IL-8 receptor, leading to increased tumor mass, increased tumor-free weight loss, and decreased muscle mass [113].

Smoking is thought to be responsible for up to 1/3 of PC cases [114,115]. The risk of PC for current smokers increases in a dose-dependent manner up to 2.7-fold for heavy smokers of more than 25 cigarettes/per day [115,116,117]. Although low (relative risk 1.48), it appears to persist even ten years after quitting [114,115] and becomes similar to that of non-smokers as late as 15–20 years after quitting [115]. Smoking cessation can reduce the frequency of PC at all ages, especially in EOPC [26].

Studying the epidemic trends of smoking, it was observed that there is a difference according to country and gender. The incidence of smoking among men in the Anglo-Saxon and North-West European countries has a wave pattern, with a peak around 1986 and a subsequent decline. In women in the same area, compared to men, the incidence started to increase after 24.2 years, on average, and peaked in 2014, reduced afterward by approximately 22.9%, but in some countries, it is still increasing. In low-income countries, the incidence peaked approximately ten years later (1993) [118]. Projection models estimate a continued decline in the smoking-associated mortality fraction (SAMF) for European men from an average of 25% in 2014 to an average of 11% in 2040, 7% in 2065, and 6% in 2100, while for women, there will initially be an increase followed by a steady decrease of 10% in 2040, 5% in 2065, and 4% in 2100, thus remaining below the average SAMF for men [119].

The incidence of EOPC was significantly increased in men in central and eastern Europe [26]. This overlaps with the gender-specific smoking prevalence rate [120]. Thus, it can be considered that individuals more susceptible to pancreatic lesions caused by the carcinogens contained in tobacco products could lower the onset of pancreatic cancer. Different studies found that EOPC patients were more frequently smokers, with a significantly younger age at smoking initiation than those with typical onset PC [23]. Multiple studies have shown that passive smoking is not associated with PC [121,122].

Although the rate of conventional cigarette smoking appears to be declining, the use of alternative nicotine-delivering products is increasing [123,124,125,126].

Regarding the use of alternative forms of nicotine products,

- Snus, a smokeless oral tobacco product primarily used in Sweden and Norway, was not linked to the development of PC, and health risks are considered to be lower than those associated with cigarette smoking [127,128]
- Electronic cigarettes are battery-operated devices that heat a liquid containing nicotine, a solvent, and one or more flavors. Studies on substances contained in e-cigarette aerosols have shown that they may contain carcinogens such as formaldehyde and acetaldehyde, but in much lower amounts than in the conventional cigarette [129,130], or supersaturated 1,2-propanediol vapor, which causes an increased production of nitric oxide, promoting inflammation [131,132], but there are still no studies evaluating the possible effect on the pancreatic tissue [133]
- Heated tobacco products generate aerosols from heating tobacco at temperatures lower than combustion temperatures [134]. It has been shown that, similar to e-cigarettes, they may contain carcinogenic substances, but at a lower level than conventional cigarettes [135]

Smoking, in addition to the fact that it supports tumor genesis and its growth, also causes a decrease in the patient’s muscle mass and weight, thus accelerating the clinical deterioration. Considering that chemotherapy can be administered depending on the Eastern Cooperative Oncology Group (ECOG) performance status, patients should be advised to quit smoking even after diagnosis, to improve nutritional status, and to decrease tumor progression. Increasing anti-smoking campaigns to reduce the number of smokers from the beginning, as well as the promotion of programs to quit smoking, could reduce the risk of cancer and other diseases that are closely related to this vice. Although, at the moment, there are different alternative options to smoking, they also do not seem safe. The long-term effects on general health and carcinogenesis are not yet fully known, as long-term studies have not yet been conducted, and their use should also be discouraged.

#### 3.2.2. Diabetes Mellitus (DM)

According to WHO, the number of people with diabetes increased from 108 million in 1980 to 422 million in 2014, and the prevalence increased faster in low- and middle-income countries than in high-income countries [136]. This is most likely due both to the fact that screening has been intensified and to the change in the definition of diabetes, with the lowering of the blood glucose level at which you are considered to have the disease.

Multiple studies demonstrated that hyperglycemia, abnormal glucose levels, and insulin resistance are associated with an increased risk of PC and also that PC has a diabetogenic effect, with half of PC patients having diabetes [137,138]. In contrast, approximately 1% of patients over 50 years old who are newly diagnosed with a metabolic disorder have PC as a trigger for their diabetes [139].

The diagnosis of type 2 diabetes (DM2) precedes that of cancer by up to 3 years in 25–85% of cases [138,140,141,142,143], thus suggesting that it may actually represent an early manifestation. Compared to the non-diabetic population, in the first year after the diagnosis of type 2 diabetes, there is a 14–15-fold higher risk of also being diagnosed with PC. The risk decreases in the second year to 3.5–5.4-fold and stabilizes at around 3-fold [144]. Diabetes could be considered secondary to the tumor-induced destruction of pancreatic acini or canalicular obstruction. However, more recent studies have shown that insulin and C-peptide secretion are increased in patients with PC and DM2, thus suggesting a more distant secondary effect on glucose metabolism, which returns to normal after cancer resection [138,145,146].

Sharma et al. developed a model called Enriching New-Onset Diabetes for Pancreatic Cancer (END-PAC) based on three factors—weight changes, blood glucose, and age of onset of diabetes—to distinguish new-onset DM (NOD) from pancreatic cancer-related diabetes (PCRD) [147]. This model has been validated in other retrospective studies [148,149,150], but not in a prospective study. A recent systematic review suggested that NOD associated with older age, a family history of pancreatic cancer, a personal history of gallstones/pancreatitis, weight loss, and a rapid increase in blood glucose is strongly related to PC and may be targeted for further screening strategies [151]. In another study, NOD associated with chronic obstructive pulmonary disease and increasing age was associated with PC [143]. High uric acid levels in diabetic women have also been associated with an increased risk of PC [152].

**Figure 4:**
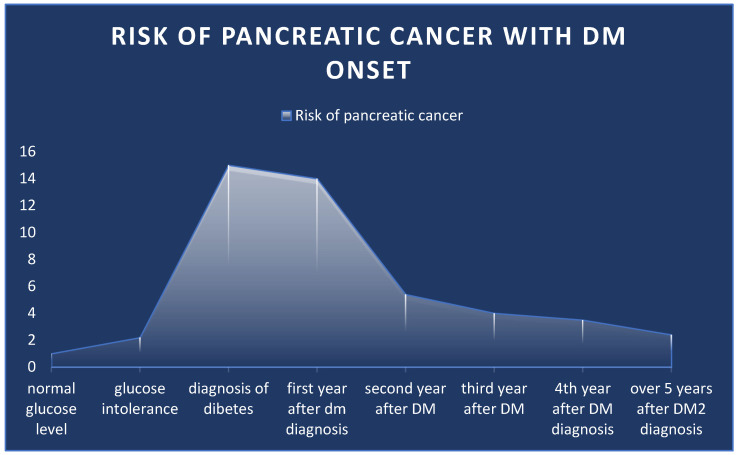
Risk of pancreatic cancer related to glucose level and onset of DM2.

In multiple prospective cohort studies, researchers have observed a 2.2-fold increased risk of pancreatic cancer among subjects with high post-load plasma glucose levels [153,154,155]. At the same time, patients with long-standing DM2 have an increased risk of up to 2.4 times (Figure 4) [156]. Regarding the risk of PC after 15–20 years of diabetes evolution, there is no consensus, as the results of the studies are inconsistent [146,156]. The mechanism that could underline the association between DM2 and PC is that insulin resistance causes compensatory hyperinsulinemia and increases insulin-like growth hormone (IGF), which stimulates pancreatic ductal carcinogenesis. Insulin promotes cell proliferation and increases glucose use [157], while IGF has mitogenic, angiogenic, and antiapoptotic activities [158,159]. A recent study found that the gene conceding the insulin receptor can also be dysregulated, and the isoform A of the insulin receptor, which also has an oncogenic function, is upregulated [160].

Regarding DM2 therapy, metformin significantly decreases the risk of PC, but interestingly, this effect was not observed in other anti-diabetics such as insulin or sulfonylurea [161,162,163,164]. Metformin acts mainly by reducing the hepatic glucose output [165], thus lowering plasma insulin concentrations and indirectly inhibiting the effects of insulin and IGF, thus having an antitumor effect, inhibiting proliferation, and stimulating apoptosis [166].

With regard to type 1 diabetes (DM1), its incidence is considerably lower than that of DM2, and there is a paucity of available data. The studies examining the association between PC and DM1 exhibit considerable variation in their definition of type 1 diabetes. Some consider a diagnosis prior to the age of 40, while others include only those who have received insulin treatment. Although the risk of PC appears to be increased up to twice that of the general population in different studies [167,168,169], the inconsistency in the definition can easily lead to confounding with type 2. Consequently, further studies are required to gain a more accurate understanding of this association.

#### 3.2.3. Obesity, Diet, and Lifestyle

The prevalence of obesity worldwide has nearly tripled between 1975 and 2016 [170]. Being overweight, with a body mass index greater than 25, increases the risk of PC, especially in women [171,172,173]. The risk appears to be increased by 20% for every 5-unit increase in BMI in early adulthood (age of 18–21 years) [31]. Obesity is linked with type 2 diabetes [174] and promotes inflammation [79,175]. Studies have shown that predominantly central abdominal adiposity distribution is more frequently associated with insulin resistance than peripheral adiposity [176]. An analysis of the pancreas in obese and diabetic individuals revealed an increased replication of the pancreatic duct by up to 10- and 4-fold, respectively, probably favoring PC development [177]. Some studies have found that a high waist-to-hip ratio may be associated with a 20–30% higher risk [28,31,178,179].

Hypercaloric diets and sedentary lifestyles lead to obesity, which causes ectopic pancreatic fat deposition and pancreatic steatosis [180]. The presence of intrapancreatic fat deposition was found to be associated with both pancreatic cancer (PC) and premalignant lesions in 52% [180], as well as with the progression of low-risk branch duct IPMN to PC [181].

Physical activity, even in the absence of weight loss, improves glucose tolerance [182]. Considering that insulin resistance increases IGF levels and stimulates pancreatic ductal carcinogenesis, having regular physical activity decreases the risk of PC. A reduced caloric intake and physical exercise have been shown to reduce the risk of PC [29].

A high-fat, high-energy diet promotes the absorption of bacterial lipopolysaccharides into circulation [183], and more lipopolysaccharide-producing bacteria have been found in the intestines of patients with PC [184]. A higher intake of red or processed meat, saturated fat, and fried foods may increase the occurrence of pancreatic cancer [185,186]. The presence of fat in the duodenum increases lipase secretion mediated by cholecystokinin-induced lipase secretion [187], thus leading to pancreatic hypertrophy and hyperplasia, increasing the risk of carcinogenesis [188].

An increased consumption of grains, nuts, vegetables, and fruits has a negative correlation with PC [185,188,189,190] and has been stated in multiple studies to even reduce the risk of PC by 38% [189], but others failed to observe such a link [191]. These foods are high in phytochemicals, such as carotenoids, phenolics, alkaloids, nitrogen-containing compounds, and organosulfur compounds [192,193], which have antioxidant and anti-inflammatory action, inhibit cell proliferation, and induce tumor suppressor gene expression and apoptosis [194]. Indeed, it was found that eating about 30 g of nuts two to three times a week and larger amounts of orange fruits and vegetables containing higher levels of beta carotene or green and red vegetables containing alpha-tocopherol is associated with a lower incidence of PC [195,196]. Isothiocyanates found in cruciferous vegetables have been shown to inhibit pancreatic cancer cells in in vitro and animal studies [197,198]. Fruits contain a large amount of vitamin C and other antioxidants that can trap free radicals, thus protecting against oxidative damage.

Regarding vitamin D levels, the data are inconsistent [199,200,201]. Vitamin D is involved in autocrine and paracrine cell differentiation, proliferation, and apoptosis [202]. 25-(OH)-D_3_ and 1,25-vitamin D analogs can inhibit cancer cell proliferation and promote apoptosis [203,204,205]. In vitro studies have shown that vitamin D regulates insulin synthesis [206]. Deficiency correlates with impaired pancreatic insulin synthesis and secretion in animals and humans [207,208]. Thus, the contradictory results highlighted by the studies can be explained at a speculative level by a possible protective effect of vitamin D on the proliferation and differentiation of pancreatic cells. Still, at the same time, an increased risk is determined by increased insulin secretion.

#### 3.2.4. Pancreatitis

Pancreatitis has a complex pathophysiology that is not fully understood. The most common etiologies of acute pancreatitis (AP) are excessive alcohol consumption and gallstone disease [209]. The development involves irregular autophagy, leading to an enhanced inflammatory response in pancreatic tissue [210].

Pancreatitis is frequently associated with PC, but similar to DM, it can also increase the risk of PC as well as develop as a result of underlying PC. The strongest correlation between acute pancreatitis and pancreatic cancer is observed in patients with AP aged 56–75 without the two common underlying causes or during the initial year following an episode of acute pancreatitis (with an effective estimate of 23.47); conversely, this correlation diminishes two years after the episode of acute pancreatitis [211,212]. The episode of AP may be, in fact, the first manifestation of PC.

After the first episode of AP, approximately 8% develop chronic pancreatitis (CP) [213]. Most cases of CP are triggered by the variable combination of environmental factors such as alcohol and nicotine consumption [213] and some genetic mutations in the PRSS1 Gene, SPINK 1, and CTFR genes [214,215].

The risk of developing PC in patients with CP is higher in patients with CP due to PRSS1 mutations [216], in those with obesity, a history of alcohol, and nicotine abuse, and in those with CP with ductal dilatation [217]. The prevailing hypothesis is that chronic inflammation leads to increased cell proliferation and decreased immunosurveillance by the downregulation of the tumor-suppressing genes p16, p53, and SMAD 4 and the upregulation of the oncogenic KRAS gene, thus favoring the onset and growth of cancer [17,79,218]. A recent meta-analysis showed that the risk of PC in CP patients increases with the duration of the disease, excluding the potential cases where PC was diagnosed in close temporal proximity to CP diagnosis [219].

Another risk of CP is being misdiagnosed as CP while having PC, which can happen in about 5% of cases [220]. Similar to DM and AP, also in the case of CP, the highest association was seen in the first two years, especially in patients between the ages of 61 and 70 years, which progressively decreased until no correlation between AP or CP with PC was observed [221], thus suggesting that the patients were either misdiagnosed from the beginning or had both CP and PC [222].

Autoimmune pancreatitis (AIP) represents a rare cause of CP. Type 1 AIP is more common and is associated with increased IgG4. It may also be associated with the involvement of other organs, such as bile ducts, salivary glands, and lungs. Type 2 AIP accounts for a maximum of 5% of AIP cases and is not associated with elevated IgG4 and other type-specific extrapancreatic manifestations, except for inflammatory bowel diseases [223]. Studies on the possible link between AIP and PC have failed to identify an increased risk for PC compared to other causes of CP, possibly due to the small number of individuals included [219,224,225,226,227]. Another explanation may be that even though there is an increase in inflammation in AIP, this may not be associated with a decrease in immunosurveillance and the downregulation of tumor-suppressor genes.

It has been observed that pancreatitis may be followed by post-pancreatitis diabetes mellitus (PPDM) in up to 80% of cases [228], which is associated with poorer glycemic control compared with DM2 [229] and also with a higher risk of developing pancreatic cancer [230].

#### 3.2.5. Alcohol

Inconsistent results have been reported for the association between alcohol consumption and PC. Some studies showed that alcohol consumption is associated with PC in a dose-dependent manner: low to moderate doses do not appear to increase the risk, while doses of >15–30–60 g of alcohol per day increase the risk up to 1.36-fold [231,232,233,234]. Other studies failed to show a link, even in genetically susceptible individuals [235,236]. Regarding the type of alcohol consumed, the data are also inconsistent. However, some meta-analyses showed that a high spirits/liquors intake was associated with an increased risk of PC in men but not in women [232,233,234]. The mechanism may be similar to tobacco use, meaning that high doses of alcohol contribute to inflammation and CP. In addition, alcohol metabolites such as acetaldehyde and even ethanol itself can cause differentiation defects in stem cells and promote inflammatory lesions and carcinogenesis by inhibiting DNA repair proteins [112,237,238].

#### 3.2.6. Coffee

The relationship between coffee consumption and pancreatic cancer is not yet clearly defined. A systematic review and meta-analysis that included 959,992 participants revealed a dose-dependent increased risk, such that each cup of coffee a day increased the risk by 1 to 6% [239,240]. On the opposite side, other prospective studies and meta-analyses have found either no association [241,242] or a decrease in risk in the same dose-dependent manner [243,244,245]. The beneficial mechanism of coffee could probably be explained by its antioxidant properties [246] and its ability to trigger tissue antioxidant gene expression and protect against gastrointestinal oxidative stress [247]. More studies are needed to determine whether and how coffee consumption is associated with PC risk.

#### 3.2.7. Hepatitis

Globally, the prevalence of hepatitis B patients is 3.5%, with higher rates in Africa and the western Pacific and the lowest rates in the USA [248]. The prevalence of hepatitis C is 1%, and the most affected regions are the Eastern Mediterranean and Europe [248]. Both hepatitis B and hepatitis C viruses are established carcinogens [249]. Although both viruses are hepatotropic and induce persistent liver injury [250,251], surface and core HBV antigens and HCV antigens have been identified in pancreatic acinar cells [252,253]. Studies have found an increased risk of pancreatic cancer among HCV- and HBV-infected patients [254,255,256]. The pathophysiological mechanism by which these viruses may contribute to PC development is not fully understood. The common origin of the blood vessels between the liver and the pancreas makes the migration of microorganisms possible [256]. The presence of these viruses in pancreatic tissue induces chronic inflammatory changes [257] that can promote proliferation.

#### 3.2.8. Gallbladder Diseases and Cholecystectomy

Gallstones are highly prevalent in the general population [258], and the standard of care for symptomatic gallstones is cholecystectomy [259]. The overall evidence for the association between gallbladder gallstones and cholecystectomy and PC is inconsistent, probably due to the study design that variably accounts for other risk factors for PC (such as obesity, diabetes, and smoking), as well as the temporal relationship between PC diagnosis and gallbladder disease. A meta-analysis showed that patients with gallstones, cholecystectomy, or both have an increased risk for PC (RR 1.7; 95% Cl 1.3–2.21, respectively; RR 1.31; 95% Cl 1.19–1.43), but the analysis was performed independently of other risk factors [260]. Some studies confirmed these findings [261,262], while others found a correlation only in the first two years after the diagnosis of gallbladder disease, suspecting that a diagnosis bias drives this short-term link, and no long-term effect was identified [263]. A large Danish cohort study including 4,465,962 individuals, who were followed up for more than 30 years, found that symptomatic gallstones and cholecystectomy were associated with a lower risk for PC, with an HR of 0.82 (0.52–1.3) and 0.32 (0.19–0.54); on the other hand, sphincterotomy was associated with an increased risk (HR 3.85 (2.23–6.63)) [264]. This increased risk may be because sphincterotomy made by endoscopic retrograde cholangiography may have an inherent translocation risk of microbial species between the oral cavity and gastrointestinal tract and the biliary system because it disrupts the anatomical and functional barriers. These changes in the microbiota may induce chronic inflammation, thus promoting carcinogenesis.

As for asymptomatic gallstones, the data show an increased risk when compared to the control group (HR 1.25; 95% Cl 0.6–2.5) [265], but with a risk similar to the control group after cholecystectomy [266].

#### 3.2.9. Periodontal Diseases

Periodontal inflammation, such as gingivitis and periodontitis, can determine systemic inflammation and is an independent risk factor for PC. A large Swedish cohort study that included over 5 million individuals found that younger individuals <50 years with mild dental inflammation, periodontitis, and root canal infection had a 35%, 56%, and 58% increased risk of PC, and those between 50 and 70 years old with periodontitis had a 20% risk of PC; meanwhile, in older patients, no association was seen [267], contrary to another study where the association between periodontal disease and PC was seen just in people aged 65 years or older (HR 2.17; 95%CI 1.03–4.57) [268]. Another study suggested that recent tooth loss could be a marker for severe periodontal disease and indirectly for PC, finding that the tooth loss over the last 4 years increased the risk for PC (RR 2.71, 95% Cl 1.70–4.32) [269]. Studies showed that *Porphyromonas gingivalis* and *Aggregatibacter actinomycetemcomitans*, two oral microorganisms often implicated in periodontitis, can increase the risk of pancreatic cancer by up to 60% also in cases without periodontitis [270,271,272,273,274]. In contrast, other microorganisms, such as Phylum Fusobacteria and its genus *Leptotrichia,* were associated with a decreased risk of PC [271]. Salivary microbiota analysis revealed decreased levels of *Neisseria elongata* and *Streptococcus* Mitis in patients with PC, with a sensitivity of 94.6% and a specificity of 82.1% in differentiating them from healthy individuals using the ROC-plot (AUC value of 0.90 (95%CI 0.78 to 0.96, *p* < 0.0001)) [275].

The underlying mechanisms between the oral microbiota and PC are not yet elucidated. However, a hypothesis may be that oral dysbiosis could determine changes in the intestinal and pancreatic microbiota that promote inflammation and oncogenesis, but further investigations are needed.

#### 3.2.10. *Helicobacter pylori* (*H. pylori*)

*H. pylori* is an established carcinogen [249], detectable in over 50% of the world’s population [276]. The association between *H. pylori* infection and the risk of pancreatic cancer remains controversial. It is primarily related to an indirect mechanism based on disrupting the cell-to-cell adhesion and intracellular signaling, triggering inflammatory processes and promoting neoplastic transformation [38,277,278,279].

#### 3.2.11. Autoimmune Diseases

Autoimmune diseases are characterized by an aberrant immune response in which immune cells react against self-antigens. While most autoimmune diseases are associated with a reduced risk of PC [280], cutaneous and systemic lupus appears to be associated with an increased risk [281] (RR 1.41 (95%Cl: 1.13–1.79), especially in the European region, but not for other regions such as America and Asia [282].

#### 3.2.12. Polycystic Ovary Syndrome (POCS)

Limited data have been reported on POCS and PC. PCOS is one of the most common endocrinological diseases affecting women of reproductive age, and it has been associated with a higher prevalence of non-alcoholic fatty pancreas, metabolic syndrome, and insulin resistance [283]. In 2019, a Swedish registry study evaluating the association between different cancer sites and PCOS reported a 3.4-fold higher risk of PC in premenopausal women with PCOS [284]. Another study also found an increased association between these two pathologies, although the association was slightly weaker after accounting for the data regarding diabetes and obesity [285].

Multiple case-control and cohort studies on women taking hormonal contraception reported no association between oral contraceptive use and the risk of PC [286,287,288].

#### 3.2.13. Microbiota

Once considered a sterile organ, recent studies established the presence of pancreatic microbiota, both in a normal and pathogenic state, but with inconsistent data regarding its role in carcinogenesis [273,289,290,291]. The possible mechanism by which the presence of different microorganisms can be involved in carcinogenesis is the ability to induce a pro-inflammatory response [292,293], inhibit the immune response aimed at eliminating tumor cells [294], and modulate cell division [279,295]. Different bacterial strains stimulate the higher release and absorption of lipopolysaccharides (LPS), which are components of the outer membrane of Gram-negative bacteria activating the host’s innate immune system [183,296], promoting chronic inflammation at the beginning, because afterward, LPS reduces the immune response by interacting with NF-kB and upregulating the expression of programmed cell death ligand 1 (PD-L1) [297]. Furthermore, more LPS could trigger Toll-like receptor 4 and thus inhibit tumor suppressor genes like PTEN and p53 [298,299]. Data emerging in recent years promote the idea that the host microbiome and the pancreas are interconnected and that the microbes included in that microbiome, such as Mycoplasma Hyorhinits, can even influence chemotherapy regimens used in PC [289,300,301]. Also, tumor microbiota may influence survival; patients with high alpha diversity (*Pseudoxanthomonas*/*Streptomyces*/*Saccharopolyspora*/*Bacillus clausii*) have significantly longer overall survival than those with low alpha diversity [302]. Fecal microbiota analysis showed that patients with PC have a significant increase in Bacteroides and Gammaproteobacteria and a reduction in butyrate-producing bacteria compared to healthy controls [184,303,304]. Animal studies have found that probiotics (containing Lactobacillus strain) can increase the efficacy of gemcitabine and may also increase the patient’s tolerance to chemotherapy [305]. Patients with positive intraoperative bile cultures for Klebsiella pneumoniae had better progression-free survival only after adding quinolone to gemcitabine and not on gemcitabine alone, suggesting that Klebsiella pneumoniae may promote chemoresistance [306].

Summarizing the data available, intestinal and tumoral microbiota and their products are implicated in both pancreatic carcinogenesis, tumor progression, and prognostics. However, further studies may lead to findings that may be applicable in clinical settings.

#### 3.2.14. Psychological Stress

The possible mechanism by which stress may be associated with an increased risk of cancer is the dysregulation of the thalamic–pituitary–adrenal axis and sympathetic nervous system and the inappropriate secretion of glucocorticoid and catecholamines [307], which can alter the cellular immune response to cancer cells.

Exposure to severe psychological stress in childhood and young adulthood, such as the loss of a parent by death, was assessed in a Swedish cohort study and associated with the early onset of PC, regardless of the age of loss [308]. Similarly, an increased risk for PC was also found in women but not in men, who experienced severe stress, such as the death of a child. The increased risk for PC became significant when considering the first five years after the loss of the child when the loss was due to suicide and when considering people with a history of psychiatric illnesses [309].

Several studies show that B-blockers, which are used for treating arrhythmia, chronic stress, and depression, have an inhibitory effect on tumorigenesis, inhibiting the damage induced by the catecholamines stimulation of adrenoreceptors in patients with PC, inhibiting the progression of PC in patients without metastasis [310,311,312,313,314].

#### 3.2.15. Renin-Angiotensin Inhibitors

Studies performed on animal models have found that angiotensin II has a pro-proliferative effect on smooth muscle [315]. Angiotensin I-converting enzyme inhibitors (ACI) that inhibit the conversion of angiotensin I to angiotensin II have an oncoprotective role by reducing oxidative stress and inflammation and by downregulating vascular endothelial growth factor, responsible for angiogenesis and nuclear factor kappa beta, which promotes the production of oncogenic proteins [316,317]. Recent cohort studies based on two populations showed a reduced risk for pancreaticobiliary cancer in patients taking ACI (HR 0.62 (0.54–0.72)) (HR 0.69 (0.53–0.9)) [318,319], while another one failed to find an association between ACI consumption and PC [320].

The use of ACI in patients with resected PC has been associated with more prolonged overall survival [321,322].

#### 3.2.16. Allergies

Several epidemiological studies and meta-analyses that examined the association between allergy history and PC risk found that allergies, especially respiratory ones, such as hay fever and animal and plant allergies, statistically significantly decrease the risk [323,324,325,326,327,328,329], but other prospective studies have failed to show an association [330].

The mechanism by which allergies could reduce the risk of PC still needs to be fully elucidated. Enhanced immune surveillance may have a role [323,331], leading to the elimination of neoplastic cells [332] by favoring the IgE response against tumor antigens [333].

While the potential role of the medical treatment of allergies in PC is an intriguing area of study, our current understanding is limited [330,334]. Further research is needed to explore this association and its implications.

#### 3.2.17. Opioids

Opioids are drugs used for pain management and recreational purposes, with increased use over the past decade. It has been shown that opioids can promote cancer progression in various cancer types by promoting angiogenesis and epithelial-to-mesenchymal transition [335] or by altering the gut microbiome [336]. Multiple studies have suggested that opium use increases the risk of PC [337,338,339,340].

#### 3.2.18. Proton Pump Inhibitors (PPI)

PPIs are, in most countries, over-the-counter medications commonly used for acid-related disorders. Two recent independent meta-analyses found that PPI use was associated with an approximately 63% increase in PC risk [341,342]. The mechanism underlying the possible link between PPI use and PC is given by the effects of PPI use, such as decreased gastric acid production and hypergastrinemia. Decreased gastric acid may promote bacterial overgrowth and nitrosamine secretion, increasing pancreatic cell proliferation [343]. Second, gastrin also has a trophic effect on epithelial cells, thus favoring the development of pancreatic neoplasia [344]. On the other hand, in healthy PPI users, a gut dysbiosis that was also associated with PC was seen, thus suggesting that the impact of PPI on microbiota may also play a role in pathogenesis [345].

#### 3.2.19. Nonsteroidal Anti-Inflammatory Drugs (NSAID)

Data on PC risk and the use of NSAIDs is inconsistent. A study including 88,378 women reported a possible increased risk of PC among the individuals who used acetylsalicylic acid regularly [346]. A more recent meta-analysis, which included approximately 258,000 participants, indicated a possible protective effect of aspirin use that increases with the frequency of administrations but failed to show a similar effect for other NSAIDs [347].

#### 3.2.20. Statins

Statins have anti-inflammatory properties, but studies suggest that their use in CP patients does not decrease the risk of PC [348].

The data regarding modifiable risk factors and potential interventions have been synthesized in the table below (Table 2).

## 4. Discussion and Conclusions

The high mortality rate of PC due to a late diagnosis emphasizes the importance of developing methods for reducing the modifiable risk factors and for helping detect resectable tumors. The main goals in improving the survival of patients with PC are decreasing the factors contributing to the appearance of this neoplasia, identifying high-risk populations, and implementing efficient and cost-effective screening methods. The present review highlights the factors associated with an increased or reduced risk of PC.

With regard to non-modifiable risk factors, the CAPS guidelines suggest that screening for multiple mutations should commence at the age of 40–50 years. The issue is that, despite being recommended in many countries, the high costs and limited accessibility make it unfeasible in practice. Furthermore, the recommendations regarding BRCA mutations assume that at least one family member has already been affected. However, 50% of those with germline BRCA mutations may not have a positive family medical history of BRCA-associated cancer. In addition to the elevated risk of pancreatic cancer in individuals with BRCA mutations, another crucial consideration is the impact on therapeutic options and prognosis. BRCA-mutated pancreatic cancers are markedly sensitive to platinum-based therapies, suggesting that testing all patients with pancreatic cancer for BRCA mutations could lead to the optimization of early therapy to improve survival.

Pancreatic cancer risk increases sharply with smoking, even in the case of a small number of cigarettes or after several years of smoking, and it decreases slowly, taking over 20 years to reach the risk of never-smokers. Thus, a campaign against conventional tobacco consumption could reduce the incidence, although studies regarding alternative nicotine delivery methods are still unclear.

Aiming for a normal BMI and a balanced diet, decreasing red meat and fat consumption, increasing physical activity and fruit and vegetable consumption, associating higher nutrient density and better diet quality with a low to moderate consumption of alcohol and coffee, and rigorous oral hygiene are wise choices that could contribute to a lower risk of PC, although the data are inconsistent.

In patients over 50–60 years of age, diabetes, gallbladder disease, and recently diagnosed pancreatitis are frequently associated with pancreatic cancer in the first three years, so additional attention for monitoring could help in early diagnosis. In the case of diabetes, screening with END-PAC could help differentiate between NOD and PCRD. At the same time, treatment with metformin should be the first treatment choice for glycemic control to reduce the risk of PC.

Last but not least, avoiding stressful events and controlling them with adequate medication and therapy could reduce the risk of PC.

A new front is changing the microbiota, but as the understanding of the mechanism of association with PC is still limited, it is clear that this area can benefit from further research. Microbiota can play a role in the early diagnosis of PC, and a new therapeutic intervention based on bacteria-related function could be generated.

Although there are screening programs for high-risk patients either due to genetics or the presence of precursor lesions, or both, they do not consider other modifiable risk factors. Smoking, for example, which is known to reduce the time of PC development by up to 10–20 years, along with other risk factors, could be included to initiate early screening in high-risk patients.

## Figures and Tables

**Table 1:** The screening recommendations for those who carry a germline mutation.

Gene Mutation	Pancreatic Cancer Risk	CAPS Consensus for Surveillance	At What Age Should Surveillance Begin? According to CAPS
BRCA1	Inconsistent data about the risk	If at least one affected FDR	
BRCA 2	Inconsistent data about the risk	If at least one affected FDR or ≥two affected blood relatives	50 years
Lynch syndrome—MLH1/MSH2/MSH6	8.6-fold increased risk	If at least one affected FDR	
FAP—APC mutation	Increased up to 4 times	No data	
Peuts–Jeghers syndrome—LKB1/STK11	132-increased risk	Regardless of family history	40 years
FAMMM with CDKN2A mutation leading to changes in the p16 protein	13 to 67-fold increased risk	Regardless of family history	40 years, and if worrisome features → EUS+/−FNA and close follow-up imaging at 3–6 months
Hereditary pancreatitis—SPINK 1 mutation/PRSS1 mutations	53-fold increased risk	Regardless of family history	40 years or 20 years after the first pancreatitis attack
PALB2		At least one affected FDR	50 years
CFTR	5.3 increased risk	No data	
Ataxia-teleangiectasia ATM mutation		At least one affected FDR	50 years

**Table 2:** PC risk factors.

Risk Factor	Impact on Risk	Studies on Risk	What Can Be Done?
Smoking	Up to 2.7-fold increase	Bosetti et al. [115] Anderson et al. [116] Lugo et al. [117]	Smoking cessation campaign Advocating for alternative nicotine-delivering products for those who do not want to quit
Diabetes	14–15-fold increased risk in the first year 2.4–5-fold increased risk in the later years	Dankner et al. [144] Bosetii C et al. [156]	Advocating for a healthy lifestyle and exercise Validating, in prospective studies, the END-PAC model or refined models (adding a family history of pancreatic cancer, a personal history of pancreatitis, COPD, and uric acid) to find the patient at increased risk and including them in screening programs Favoring the use of metformin as the first-line anti-diabetic treatment
Obesity, diet and lifestyle	20–30% increased risk	Berrington de Gonzáles et al. [28] Genkinger et al. [31] Stolzenberg-Solomon RZ et al. [178] Arslan et al. [179]	Advocating for a normal weight, physical activity, and a diet rich in antioxidants such as fruits, nuts, and vegetables, avoiding the use of high amounts of meat and large amounts of soft drinks
Pancreatitis	Up to a 24-fold increased risk	Ikeura et al. [225] Hart et al. [226]	Avoiding excessive alcohol consumption Screening for patients with AP aged 57–75 years old, without the two most common ethologic factors of acute pancreatitis Screening for genetic mutations in those with CP and then further screening according to guidelines
Alcohol	Up to 1.36-fold increased risk	Tramacere et al. [231] Wang et al. [232] Gapstur et al. [233]	Avoiding excessive alcohol consumption
Coffee	Inconsistent data	Li et al. [239] Nie et al. [240] Zhou et al. [243] Ran et al. [244] Lukic et al. [245]	Moderate consumption until further studies are conducted
Viral hepatitis		Huang et al. [254] Hassan et al. [255] Xu et al. [256]	Vaccination against Hepatitis B virus, treatment for hepatitis C virus
Gallbladder diseases and cholecystectomy	Inconsistent data	Fan et al. [260] Luo et al. [261] Rosato et al. [263] Shabanzadeh et al. [265]	Promovate
Periodontal diseases		Jingru Yu et al. [268] Chang et al. [268] Michaud et al. [269]	Good oral hygiene
*Helicobacter Pylori*	Controversial data	Risch HA et al. [38] Huang J et al. [277] Bulajic M et al. [278] Cullin N et al. [279]	Promoting a balanced diet
Autoimmune diseases	Systemic and cutaneous lupus may be associated with an increased risk, but the data are controversial	Zhang M et al. [282]	A special focus on patients with autoimmune diseases and their treatment
POCS	3.4-fold increased risk	Peeri et al. [285] Yin et al. [284]	Considering the correlation of POCS with obesity and glucose intolerance; perhaps a balanced diet could influence both conditions
Microbiota	Inconsistent data	Pushalkar S et al. [289] Liu J et al. [298] Santoni M et al. [299]	Further studies needed
Psychological stress	Increased risk	Kennedy B et al. [308] Huang et al. [309]	Psychological and/or psychiatric support for very stressful events
Renin-angiotensin inhibitors	May reduce the risk of pancreatic cancer—further data needed	Lee et al. [318] Tse et al. [319]	With a doubtful effect, promoting the use of ACI for those who need antihypertensive treatment
Statins	No effect on PC risk	Kirkegård et al. [348]	Not appliable
Allergies	May reduce the risk	Gandini et al. [323] Cotterchio M et al. [324] Wang et al. [325] Gandini et al. [323] Karim et al. [329]	Not appliable
Opioids	May increase the risk	Shakeri et al. [337] Moossavi et al. [338] Barlass et al. [339] Sun et al. [340]	Limiting the use of opioids; anti-drug campaigns
NSAID	Inconsistent data	Schernhammer ES [346] Zhang et al. [347]	Inconsistent data
PPI	63% increased risk	Poly et al. [341] Zhang et al. [342]	Limiting the use; use just for those who need it

## Data Availability

No new data were created or analyzed in this study.

## Abbreviations

ACI	Angiotensin I-converting enzyme inhibitors
ACR	American College of Radiology
AGA	American Gastroenterological Association
AP	Acute pancreatitis
APC	Adenomatous polyposis coli
BD-IPMN	Branch duct intraductal papillary mucinous neoplasm
BMI	Body mass index
CA 19-9	Cancer antigen 19-9
CAPS	International Cancer of the Pancreas Screening
CEA	Carcinoembryonic antigen
CP	Chronic pancreatitis
DM	Diabetes mellitus
DM1	Diabetes mellitus type 1
DM2	Diabetes mellitus type 2
DNA	Deoxyribonucleic acid
ECOG	Eastern Cooperative Oncology Group
END-PAC	Enriching New-Onset Diabetes for Pancreatic Cancer
EOPC	Early onset pancreatic cancer
EUS	Endoscopic ultrasound
FAMMM-PC	Familial atypical mole melanoma pancreatic carcinoma syndrome
FAP	Familial adenomatous polyposis
FDR	First-degree relative
^68^Ga-FAPI PET PET	using ^68^Ga-labeled fibroblast activation protein inhibitor
HBV	Hepatitis B virus
HCV	Hepatitis C virus
HNPCC	Hereditary nonpolyposis colorectal cancer (Lynch syndrome)
IGF	Insulin-like growth factor
IPMN	intraductal papillary mucinous neoplasm
KRAS	Kirsten-rat sarcoma
LOPC	late-onset pancreatic cancer
LPS	Lipopolysaccharides
MCN	Mucinous cystic neoplasm
MD-IPMN	Main duct intraductal papillary mucinous neoplasm
MPD	Main pancreatic duct
MRCP	Magnetic resonance cholangiopancreatography
MRI	Magnet resonance imaging
NOD	New-onset diabetes mellitus
NSAID	Nonsteroidal Anti-Inflammatory Drugs
PC	Pancreatic cancer
PCN	Pancreatic cystic neoplasm
PCRD	Pancreatic cancer-related diabetes
PD-L1	Programmed cell death ligand 1
PET	Positron Emission Tomography
PJS	Peutz-Jeghers syndrome
POCS	Polycystic Ovary Syndrome
PPDM	Post-pancreatitis diabetes mellitus
PPI	Proton Pump Inhibitors
SAMF	smoking-associated mortality fraction
SDR	second-degree relative
SEER	Surveillance, Epidemiology and End Results
STK	Serine-threonine kinase
WHO	Word Health Organization
